# Cost-benefit analysis: the first real rule of fight club?

**DOI:** 10.3389/fnins.2013.00248

**Published:** 2013-12-19

**Authors:** Kristin L. Hillman

**Affiliations:** Department of Psychology, University of OtagoDunedin, New Zealand

**Keywords:** competitive behavior, decision making, aggression, cost-benefit, utility, competition

## Abstract

Competition is ubiquitous among social animals. Vying against a conspecific to achieve a particular outcome often requires one to act aggressively, but this is a costly and inherently risky behavior. So why do we aggressively compete, or at the extreme, fight against others? Early work suggested that competitive aggression might stem from an innate aggressive tendency, emanating from subcortical structures. Later work highlighted key cortical regions that contribute toward an instrumental aggression network, one that is recruited or suppressed as needed to achieve a goal. Recent neuroimaging work hints that competitive aggression is upmost a cost-benefit decision, in that it appears to recruit many components of traditional, non-social decision-making networks. This review provides a historical glimpse into the neuroscience of competitive aggression, and proposes a conceptual advancement for studying competitive behavior by outlining how utility calculations of contested-for resources are skewed, pre- and post-competition. A basic multi-factorial model of utility assessment is proposed to account for competitive endowment effects that stem from the presence of peers, peer salience and disposition, and the tactical effort required for victory. In part, competitive aggression is a learned behavior that should only be repeated if positive outcomes are achieved. However, due to skewed utility assessments, deviations of associative learning occur. Hence truly careful cost-benefit analysis is warranted before choosing to vie against another.

A critical consideration in social decision-making is whether or not to compete against a conspecific. Competitive action can take many forms, for example it could involve a quick, direct, physical fight between two individuals, or long, covert, strategic manoeuvres between groups. In all of its forms, competitive engagement carries the implicit goal of outperforming conspecifics in order to achieve resources or other outcomes that facilitate self-preservation. Direct competitive aggression is one of the most observable forms of competitive engagement amongst social animals. This specific form of competitive action is frequently required to obtain or protect a resource, but it is energetically costly and inherently risky. So how do we know when (or when not) to put up a fight?

Early investigations in psychoanalysis, ethology and neuroscience suggested that animals have an innate aggressive drive, stemming from basally active subcortical networks. While this idea might explain the behavior of certain characters from the 1990's media phenomenon *Fight Club*, it does not fit well with common patterns of animal behavior. Socially, constant aggressive tendencies would create a tense and nihilistic world. Physiologically, subcortical circuits that were basally active would require an inordinate amount of cortical energy to suppress. It would be more evolutionarily advantageous for animals to have an instrumental aggression network (IAN), one that can be recruited for competitive action only when it's worthwhile to compete.

Determining whether competitive aggression is worthwhile represents a cost-benefit decision, largely reliant on the same neural networks that process non-social decision variables. There is an outcome at stake that you want. How much do you want it and what costs will be incurred in obtainment? Reward valuation and cost assessment are paramount. The presence of others who also want that same outcome simply makes for multi-factorial cost-benefit analysis. Peer interest should enhance the utility of the outcome, providing an endowment effect that can be modulated by the composition of the peer group, and the expected ferocity of their competitive tactics. Expended competitive effort can discount the utility of the outcome, but can also provide an immediate endowment effect of deservingness for the victor. Multi-factorial cost-benefit analysis thus structures competitive aggression, informing us when and when not to put up a fight.

## An innate drive to fight?

In the 21st century it's easy to ascribe competition to supply-and-demand; in increasingly crowded environments, resource competition is mathematically inevitable. But perhaps there is something more basic, more primal occurring. Perhaps there is an innate need to, at times, be agonistic and aggressive toward others? Lorenz termed this the fighting instinct (Lorenz, 1966), Freud summed it up as the outward expression of the internal death drive: *thanatos* (Freud, 1922).

Goltz (1892) and others in the late 19th and early 20th centuries started to give neural credence to this idea of an innate aggressive drive. Decerebrate dogs and cats exhibited abnormally aggressive behavior, spontaneously and in response to non-noxious stimuli such as routine handling (Goltz, 1892; Bard, 1928, 1934). The emergent idea that aggression stemmed from subcortical structures was strengthened by early stimulation studies. Subcortical stimulation, specifically in the posterior hypothalamus, produced agonistic behavior in birds and cats (Woodworth and Sherrington, 1904; Ingram et al., 1932; Bard, 1934; Hess and Brugger, 1943; Hess, 1954; Holst and St. Paul, 1960; Phillips and Youngren, 1973). This “sham rage” incorporated a range of phenotypic combative behaviors (Cannon and Britton, 1925; Bard, 1934). Sano et al. (1970) were the first to use electrocauterization of the posterior hypothalamus in humans to successfully reduce pathological aggression.

In addition to the posterior hypothalamus, regions of the brain stem and thalamus have been found to contribute toward sham rage responses. For example, stimulation of the periaqueductal gray (PAG) can elicit aggressive behaviors, vocalizations and lowered fear responses in a variety of species (Magoun et al., 1937; Kelly et al., 1946; Delgado, 1963; Phillips and Youngren, 1973). Lesioning of the PAG prevents hypothalamus-stimulated sham rage from occurring, indicating a functional coupling between these regions in aggressive behavior (Fernandez De Molina and Hunsperger, 1962). Lesions to the locus coeruleus also result in submissive behaviors in rats when competing for water (Plewako and Kostowski, 1984). Manipulations to the ventral thalamus, the diencephalic extension of reticular activating system, mimic brain stem manipulations. Stimulation of ventral thalamus in monkeys results in antisocial, fighting behavior (Delgado, 1963), whereas lesioning results in behavioral inhibition in rats (Turner, 1970), cats (Adey et al., 1962), and humans (Andy et al., 1963).

Thus areas of the posterior hypothalamus, midbrain and ventral thalamus contribute toward an aggression network, with electrical stimulation of any node of the network resulting in sham rage. Baseline activity within the network—usually suppressed by higher level cortical mechanisms—could represent a primal, *thanatos*-like drive to dominate conspecifics. In decorticate animals sham rage was sometimes reported to occur spontaneously, indicative of basal subcortical activity (Goltz, 1892; Bard, 1928, 1934). However, such spontaneous rage was often directed toward non-specific objects and sometimes even self-directed. Hence basal activity in this subcortical aggression network is unlikely to drive strategic competitive aggression; the resultant actions do not enhance, and could actually hurt self-preservation, the ultimate evolutionary goal of competitive action.

## Instrumental aggression network

In decorticate animals sham rage was more often reported in response to stimuli, both noxious and non-noxious stimuli. This suggests that cortical mechanisms, instead of constantly suppressing a basally active subcortical network, serve to activate an aggression network in response to incoming stimuli. Regions in the hypothalamus, brain stem, and ventral thalamus could therefore be said to contribute toward an IAN. In corticate animals, the IAN is recruited when sensory stimuli indicate that aggressive action is instrumental toward self-preservation. In decorticate animals, appropriate assessment of what constitutes aggression-inducing sensory stimuli is lacking, and sham rage can result.

Assessment and valencing of sensory stimuli as aggressive-inducing or otherwise implies a role for the amygdala, and indeed stimulation of the amygdala produces defensive reactions that have been interpreted as sham rage (Clemente and Chase, 1973). However such behavior is ameliorated by hypothalamic or midbrain lesion (Fernandez De Molina and Hunsperger, 1962), suggesting that “amygdaloid rage” is dependent on downstream activation of hypothalamic or midbrain nodes of the IAN. Amygdaloid lesions result in loss of competitive behaviors in dogs, cats and rodents when competing against conspecifics for food (Fuller et al., 1957; Bunnell et al., 1966; Zagrodzka et al., 1983; Lukaszewska et al., 1984). Lesions of the amygdala in monkey can result in a loss of social dominance (Rosvold et al., 1954) or generalized placidity (Kluver and Bucy, 1939). Stereotactic amygdalotomy has been used successfully in humans to treat intractable aggression (Mpakopoulou et al., 2008).

In some studies, however, amygdaloid lesions have produced the opposite effect on aggression. Bard and Mountcastle (1948) and Wood (1958) reported that ablation of the amygdala in cat produced an increase in aggression. Elements of the Kluver and Bucy (1939) also hint at contradictory patterns of behavior: amygdalotomy in monkeys produces general placidity, yet hyperactivity, hypersexualty, and hyperreactivity to environmental stimuli. Behavioral differences in amygdaloid lesion studies are likely attributable to spatially distinct functional regions within the structure.

With regard to the IAN, stimulation of the basolateral amygdala (BLA) increases hypothalamic excitability while stimulation of the corticomedial amygdala (CMA) suppresses hypothalamic discharge (Dreifuss et al., 1968). Further studies that specifically targeted the stria terminalis, the major septal pathway linking the CMA to the hypothalamus, showed that electrical stimulation of this pathway inhibits aggression in monkeys (Delgado, 1963), while destruction of this pathway increases aggression and dominance in rodents and cats (Brady and Nauta, 1953; Fernandez De Molina and Hunsperger, 1959; Turner, 1970). The central nucleus of the amygdala projects inhibitory afferents to nodes of the IAN, including the hypothalamus and brainstem (Jongen-Relo and Amaral, 1998; Saha et al., 2000; Ghashghaei and Barbas, 2002).

Findings such as these suggest the CMA and its major subcortical afferent pathway play an important role in braking immediate IAN activation upon sensory input. This initial braking mechanism may be overruled by dangerous stimuli (e.g., pain), which near-reflexively activate the sympathetic nervous system and the thalamo-amygdala pathway. This can prompt IAN activation and subsequent aggression. Indeed aggression is frequently observed in response to painful stimuli, providing a feedforward mechanism for escalation of aggression in combative fights.

Alternatively, this CMA braking mechanism on the IAN may be potentiated by fear- or caution-inducing stimuli (e.g., vocalizations from dominant conspecifics), which would contribute toward the efficacy of threat cues in preventing competitive fights. In rats, CMA lesion results in failure to avoid dominant conspecifics (Luiten et al., 1985). In humans, increased amygdalar activity is observed in response to fearful facial expressions (Asghar et al., 2008; Gamer and Buchel, 2009), however, reduced amygdalar activation is seen in the same task in children with disruptive behavioral disorders (Marsh et al., 2008; Jones et al., 2009). This contrasts with reports of increased amygdalar activation in response to social threat cues in individuals with impulsive aggression (Coccaro et al., 2007). These contradictions may speak to a functional separation between the CMA and the BLA that, in the past, has been difficult to resolve with neuroimaging. Newer approaches though, for example the functional connectivity MRI seed analysis used by Bickart et al. (2012), are starting to delineate regional differences within the amygdala in regard to social behavior.

In opposition to the IAN braking mechanism exerted by the CMA, activity in the BLA can enhance activity in subcortical IAN nodes (Dreifuss et al., 1968). Given the BLA encodes incentive value of stimuli across time (Pickens et al., 2003; Holland and Gallagher, 2004; Winstanley et al., 2004), highly salient sensory stimuli—positively or negatively valenced—may drive IAN activation, spurring aggressive behavior. This could account for the emergence of competitive aggression to obtain highly appetitive resources, or frustration aggression after a salient, negatively valenced event such as the absence of an expected reward. Amygdalar hyperactivity is reported in instances of reactive “hot” aggression and other forms of impulsive behavior (Coccaro et al., 2007; Sterzer and Stadler, 2009). It is possible that BLA activity accounts for the majority of this amygdalar hyperactivity seen in reactive “hot” aggression studies, with BLA activity driving IAN activity, resulting in combative behavior. Again approaches such as functional connectivity MRI seed analysis (Bickart et al., 2012) could be used to test interactions between the BLA and the IAN, and the CMA and the IAN, in relation to aggressive behavior.

In the search for the common denominator of amygdala function—e.g., valence, arousal, or relevance—competitive activation may be worth considering. Amygdalar assessment of sensory stimuli could inform an organism to “act now, act competitively” or to “not act competitively in this situation,” keeping in mind that *acting competitively* encompasses a range of tactics. For example, one may need to act quickly (scramble competition), aggressively (contest competition) or slyly (strategic competition). In line with this idea, abnormalities in amygadalar activity would manifest as impaired competitive effort allocation, generating a spectrum of behaviors ranging from hyperaggression on one end, to avolition and social withdrawal on the other. A similar spectrum is seen following damage to regions of the prefrontal cortex (PFC). Blumer and Benson's characterization (1975) of pseudopsychopathy and pseudodepression, correlated to damage in the orbitofrontal cortex (OFC) and dorsolateral PFC (dlPFC), respectively, could also be framed as deficits in competitive effort allocation, and suggest that the PFC also plays an important role in modulating competitive action.

## Prefrontal modulation of aggressive behaviors

Advanced oversight of competitive aggression, particularly in terms of preventing actions that could prove costly, is usually attributed to the PFC. The ventromedial PFC (vmPFC), OFC, anterior cingulate cortex (ACC) and dlPFC have been implicated in controlling aggressive behaviors. Prefrontal regulatory control over the IAN can occur via direct pathways to the subcortical nuclei or via indirect pathways utilizing the amygdala (Ongur et al., 1998; McDonald et al., 1999; Delville et al., 2000; Etkin et al., 2006; Toth et al., 2010). In humans, activity in the vmPFC decreases when subjects imagine aggressive actions (Pietrini et al., 2000), and hypoactivity in the OFC and ACC is reported in aggressive cohorts (Davidson et al., 2000). OFC hypoactivity is seen in manic phases of bipolar disorder (Blumberg et al., 1999), and in borderline personality disorder (Soloff et al., 2003). Damage to the OFC produces a well-established dysregulation of behavior which can include aggressive outbursts and impulsiveness (Anderson et al., 1999). PFC hypoactivity, coincident with hyperactivity in the amygdala, midbrain and thalamus, was reported in a PET study of criminals who committed impulsive/affective murders (Raine et al., 1997).

In laboratory animals, OFC lesions variably affect aggression (Giancola, 1995), in part due to complicated bidirectional connectivity with the amygdala. Caudal OFC sends a direct projection to the central nucleus of the amygdala, activation of the latter serving to inhibit hypothalamic and brainstem regions of the IAN (Ghashghaei and Barbas, 2002). However, OFC also sends projections to the intercalated masses of the amygdala, where excitation of local GABAergic cells inhibit central nucleus output (Ghashghaei and Barbas, 2002), which would disinhibit the IAN. Hence OFC is poised to both recruit and suppress competitive aggression.

The ACC, dlPFC, and vmPFC are more implicated in suppressing aggressive behaviors. In cats, bilateral lesion of the ACC gyrus generates a hyperaggressive phenotype, inclusive of sham rage in response to handling and directed rage toward conspecifics (Kennard, 1955). Stimulation of the ACC gyrus or dlPFC increases the latency and reduces the severity of hypothalamic-induced feline sham rage (Siegel and Chabora, 1971). In monkeys, bilateral ablation of the dlPFC increases aggression (Kamback and Rogal, 1973; Mass and Kling, 1975).

In humans, dlPFC activation is seen in many instances of emotional regulation, some instances perhaps necessitating suppression of a desire to act combatively toward a conspecific, e.g. accepting unfair offers in the Ultimatum game (Sanfey et al., 2003). The dlPFC, OFC, and ACC are also activated when people are intentionally angered (Dougherty et al., 1999; Kimbrell et al., 1999) or shown angry facial expressions (Blair et al., 1999), but withhold reactive behaviors. This emotional regulation may be analogous to reversal learning, whereby one is suppressing aggressive output in response to stimuli which may have previously aroused negative affect (Davidson et al., 2000).

This type of emotional regulation, whereby aggressive reactions are suppressed, has implications for social hierarchy maintenance, which in turn influences competitive behavior. While direct competitive aggression is needed to initially establish a hierarchy, dominance hierarchies ultimately serve to reduce fighting amongst social animals. Growing evidence suggests that the dlPFC and ACC register elements of social state that may then modulate downstream activation of the IAN. For example, Fujii et al. (2009) reported that neurons in monkey dlPFC register social state during a competitive food-grabbing task, with neurons of dominant monkeys in an “up state” and neurons of submissive monkeys in a “down state.” Wang et al. (2011) reported that neurons in the ACC and prelimbic cortex of dominant mice exhibit heightened AMPA-mediated synaptic efficacy as compared to subordinate mice. Moreover molecular manipulations that increased or decreased medial prefrontal synaptic efficacy in these mice resulted in respective upward or downward movements in social rank (Wang et al., 2011).

One interpretation of these studies is that heightened tonic prefrontal activity in dominant animals may indicate that the network is “primed” for action, and aggressive tactics—via downstream activation of the IAN—can be deployed quickly if needed. Quick aggressive responses would increase the chances of success in a competitive encounter and thereby maintain social rank. In this way tonic prefrontal activity may be more indicative of behavioral planning, as compared to the phasic prefrontal activity patterns that are linked to acute inhibition of aggressive behavior and cognitive control of emotion (Miller and Cohen, 2001). Indeed the vmPFC, OFC, ACC, and dlPFC are poised to drive aggressive tactics if instrumental in achieving a desired outcome. Reward encoding is well-established in the OFC (Schoenbaum et al., 2000; Wallis and Miller, 2003; Walton et al., 2007), social reward encoding in the ACC gyrus (Rudebeck et al., 2006; Chang et al., 2013), effort-outcome encoding in the ACC (Walton et al., 2007; Hillman and Bilkey, 2010, 2012), and subjective value is represented in the vmPFC (Kable and Glimcher, 2007). Thus, depending on which literature is followed, these prefrontal regions comprise an emotional regulation network or a reward-based decision-making network.

Parsimony can emerge between the two when competitive aggression is viewed in terms of cost-benefit analysis: Is an aggressive action/emotional reaction worthwhile? Prefrontal activity can suppress combative behaviors if they are likely to be costly to the individual, or drive competitive tactics if beneficial. Indeed justified aggressiveness (e.g., attacking an attacker) is associated with PFC activation in humans and rats (Halasz et al., 2006; King et al., 2006). In humans, simply viewing a superior ranked competitor elicits activity in the PFC, amygdala and thalamus (Zink et al., 2008)—perhaps readying, and/or steadying, IAN activation.

## Deciding to compete

If the IAN is not basally active, and in fact oftentimes purposely suppressed when angered, then what drives recruitment? Other than in pathological conditions of non-instrumental aggression, we compete with each other only when it's worthwhile, i.e., the outcome of pending competitive aggression is deemed valuable. Tangible resources that aid self-preservation could be in question, or self-preservation itself might be the goal in situations of self-defence. Cost-benefit-based outcome valuation thus becomes the lynchpin of competitive action: if there is not something worth fighting for, then you won't fight.

It is well-established that in non-social choice behavior, outcome valuation is learned via trial-and-error and is dependent on midbrain-striatal-frontal circuitry. Phasic activation of midbrain dopaminergic cells correlates to reward prediction errors (Schultz, 1998). Downstream activity in ventral striatum occurs in both appetitive and aversive learning paradigms, with dorsal striatum implicated in response-reward contingencies (Schultz et al., 2003; O'Doherty et al., 2004; Cohen, 2008). Primary and secondary reward preferences, reward anticipation and reward receipt have been correlated to single-unit and fMRI activity in the OFC (Critchley and Rolls, 1996; Watanabe, 1996; Gottfried et al., 2003; Small et al., 2003; Kennerley and Wallis, 2009). When incurred costs need to be integrated with reward value, various prefrontal subregions are recruited (Walton et al., 2007; Hillman and Bilkey, 2010). Negative outcomes elicit consistent activity in the anterior insula (AI) proportional to subjective aversion (Mojzisch and Schulz-Hardt, 2007; Seymour et al., 2007). The intensity of an outcome, irrespective of positive or negative valence, has been linked to activity in the amygdala (Holland and Gallagher, 2004). Together these regions provide an assessment of outcome, pre- and post-action, that help to optimize non-social choice behavior over time.

It is plausible that these same regions provide an assessment of outcome, pre- and post-competitive aggression, that help to optimize competitive behavior over time. Social actions, competitive or otherwise, have positive or negative outcomes for the self, which may be better or worse than expected. Social actions that enhance the evolutionary fitness of an individual should be represented as “rewarding,” e.g., positive prediction errors in midbrain-striatal regions would be expected, as well as increased activity in OFC for preference formation. Social actions that hamper an individual's fitness should be represented as “aversive,” e.g., activity in AI would be expected proportional to negative affect, as well as increased activity in ACC for unrequited effort and conflict. In line with Thorndike's Law of Effect (1911) and reinforcement learning theory (Sutton and Barto, 1998), any social course of action that results in a self-referenced positive outcome should be increasingly repeated.

Winning a direct competitive encounter does reinforce competitive behavior across a variety of species. For example, victorious fruit flies are more likely to instigate subsequent competitive bouts, with markedly higher odds of victory in that bout (Chen et al., 2002; Yurkovic et al., 2006). In humans, winning a competitive encounter elicits activity in the ventral striatum and OFC, even if winning is passively achieved (Katsyri et al., 2013; van den Bos et al., 2013; though see Delgado et al., 2008). Winning against a superior-ranked player additionally elicits activity in the dorsal striatum, mPFC and nodes of the IAN, suggesting establishment of a profitable, aggression-dependent action-outcome contingency (Zink et al., 2008).

In humans, winning also activates the temporoparietal junction (TPJ). Win-related TPJ activation is greater when a larger reward is at stake (Halko et al., 2009), and also greater in subjects who attribute higher utility to winning in self-report measures (van den Bos et al., 2013). Functional connectivity between the TPJ-ventral striatum/-vmPFC is predictive of overbidding behavior in a competitive auction task (van den Bos et al., 2013), perhaps indicative of salience reinforcement. TPJ is implicated in theory-of-mind and mentalizing networks (Assaf et al., 2009), and also in directional attention (Corbetta et al., 2008; Mitchell, 2008). Given modern society's emphasis on the importance of winning, it is possible that winning—no matter how menial or inconsequential the competitive testing task—drives directional attention which accounts for this TPJ activation. This would account for modulation of TPJ activity in relation to reward size (Halko et al., 2009) and personal attribution (van den Bos et al., 2013). Parcellation of TPJ subregions, as has been recently shown by Mars et al. (2012), represents an important step forward in delineating the variable functions of the TPJ in social and non-social settings.

While winning reinforces competitive behavior, losing results in progressive extinction of competitive behavior across a variety of species. For example, defeated rodents exhibit defeatist behavior in subsequent competitive encounters, and show rapid extinction in race running (Kahn, 1951; Kanak and Davenport, 1967). Defeated fruit flies develop a “loser's mentality” (Yurkovic et al., 2006). Human data is varied; while psychosocial research provides evidence of defeatist patterns of behavior (e.g., related to oppression), laboratory studies of competition often indicate behavioral activation following a defeat. For example, losing in a starting round of an iterative competitive auction reliably prompts overbidding in subsequent initial rounds (van den Bos et al., 2013). One important distinction between datasets is that repeated encounters/sessions are required for behavioral extinction, not just repeated trials within a single encounter/session. Neuroimaging studies examining repetitive sessions between the same opponents would be of interest.

Neurally, losing a competitive round prompts activation of the ventral striatum, AI, dorsal ACC, and nodes of the IAN in humans (Delgado et al., 2008; Zink et al., 2008; van den Bos et al., 2013). Negative prediction errors in the ventral striatum occur alongside subjective aversion signals in AI and signals of conflict, unrequited effort or perhaps even social pain (Eisenberger et al., 2003) in dorsal ACC. Activation of the IAN should drive a subject to perform more aggressively in a subsequent round in an attempt to win. AI activity increases when losing to inferior-ranked players (Zink et al., 2008) and in subjects who attribute greater aversion to loss in self-report measures (van den Bos et al., 2013). Functional connectivity between the AI-ventral striatum/-vmPFC predicts trial-by-trial overbidding in auction tasks (van den Bos et al., 2013). Functional connectivity between the AI and the OFC predicts defection by a player following a non-reciprocated exchange in the Prisoner's Dilemma Game (Rilling et al., 2008). After a subjective loss, signals from the AI appear to be important in updating striatal and prefrontal utility estimates, helping to drive subsequent vigor in some instances, or withdrawal in others.

## A multi-factorial calculation of utility

Competitive behaviors seem to be driven by more than sheer resource value, given that we pursue resources differently depending on if we're alone, if we're amidst friends, or if we're amidst enemies. This suggests that utility estimates of desired resources/outcomes are different in social settings. To build a simple model of this altered utility estimate, assume that in a non-competitive scenario, a desirable resource holds a utility (*U*) of *x*; *x* being a value greater than zero, representative of a cost-benefit valuation that has been previously established via trial-and-error and/or observational learning. Chang et al. (2013) have recently shown that, in social settings, prefrontal subregions differentially encode resource valuations (*x*) based on frame of reference. Self-referenced valuations predominate in the OFC and ACC sulcus, with the former sensitive to self-experienced rewards and the latter to self-experienced foregone rewards. Other-referenced valuations predominate in the ACC gyrus (Chang et al., 2013), a region previously shown to be important in conspecific-based learning (Behrens et al., 2008).

In a competitive social scenario, the resource still holds a value of *x*, however, now others also want this resource. This produces a pre-obtainment endowment effect: the resource's value increases, *U* = *x* + (*x* * *a*_1_), where *a*_1_ represents peer interest endowment and ranges from 0 to 1. No peer interest in the resource (*a*_1_ = 0), up to high peer interest in the resource (*a*_1_ = 1) modulates the perceived utility of the resource which can prompt action. Alternatively, if peer disgust is exhibited for the resource, −1< *a*_1_ < 0, decreasing utility and dissuading action.

Behaviorally, the mere presence of others does prompt resource scavenging, as is seen in social facilitation of feeding. Satiated animals will start eating again when new animals arrive and start eating (Bayer, 1929; Harlow, 1932). Rats trained to press levers at 10 s intervals for food will become impulsive in the presence of other rats, pressing the lever before the 10 s interval (Wheeler and Davis, 1967). Both scenarios could be interpreted as scramble competition; the resource has enhanced value in the presence of others, which spurs action. Neurally, the presence of others alters reward-related activity in the ventral striatum and OFC during resource-based tasks. For example, when humans decide to donate money to charity or keep it for themselves, the mere presence of an observer increases activity in the ventral striatum during the decision phase (Izuma et al., 2010), perhaps reflective of a peer interest endowment (*a*_1_) of the monetary value (*x*). Likewise, Azzi et al. (2012) have recently shown that when fluid-deprived monkeys complete a task to receive a medium sized drop of water (*U* = *x*), the mere presence of a conspecific effectively doubles single-unit encoding of reward value in the OFC, perhaps reflective of *U* = *x* + (*x* * *a*_1_).

Peer interest endowment (*a*_1_) is further modified by the composition of the peer group (*g*, where 1 < *g* < 2) and their anticipated aggressiveness (*y*, where *y* = 1 or −1), such that *U* = *x* + (*x* * (*a*_1_ * (*g* * *y*))). A congenial group of competitors who also want the resource (*g* = 1) would exert no further change in utility beyond the initial peer endowment effect (*a*_1_). A group of established adversaries who also want the resource (*g* = 2) would effectively double the peer endowment effect, trebling utility from the initial intrinsic value *x*. However, even if a resource has become highly valuable due to an adversary's interest in the resource, impulsive action would be unwise without weighing in potential losses that might soon occur, in terms of lost effort, lost status or even loss of life. If anticipated competition is expected to be fair, with acceptable, proportional costs, *y* = 1. However, if anticipated competition is expected to be highly aggressive and contentious, for example against an established dominant conspecific, *y* = −1. Hence utility assessments would be highest for a fair fight against adversaries, and lowest for an anticipated unfair/hostile fight against adversaries.

When considering situations where animals don't compete for a resource—they submit—the largest determinant appears to be hierarchy. In a series of experiments in the 1960s, work by Delgado (1966, 1967) showed that sham rage in monkeys—elicited by stimulation of the ventral thalamus or PAG—was modulated by previously established social hierarchy. Sham rage inductions in high-ranking males did not prompt the males to attack their companion females, however, the males showed targeted aggression toward monkeys with whom a past conflict had occurred. Sham rage inductions in low-ranking males, when in isolation, produced the usual repertoire of aggressive behaviors; however, subcortical stimulation carried out in the presence of a conspecific would produce fleeing behavior in these monkeys. These studies provided an initial indication that even when the IAN is exogenously activated, learned higher-level *g* and *y* components can influence competitive engagement.

Recent work in monkey by Santos et al. (2012) has highlighted a subpopulation of neurons in caudate nucleus that may contribute toward the *g* and *y* components termed herein. These social state *S* neurons appear to encode social state dynamics during competitive food-grabbing tasks. *S* neurons have highest activity when reward grabbing is uncontested, and lower activity when monkeys act submissively due to a competitor's behavior (Santos et al., 2012). When combined with reward-related outcome information that is encoded in the caudate by a separate subpopulation of reward *R* neurons, the resultant signal should help adjust competitive behaviors in dynamic social contexts (Santos et al., 2012). In human imaging studies, caudate activity has been shown to increase when subjects cooperate with each other (Rilling et al., 2002), perhaps indicative of a *g* = 1/*y* = 1 situation where the objective is uncontested, and *S* neuron activity should be high.

The ACC, vmPFC, and OFC also likely contribute toward peer-related valuations (*g, y*) prior to competitive encounters, as these regions are sensitive to conspecific assessment in non-competitive tasks. For example, in a human imaging study by Behrens et al. (2008), participants in a choice task were challenged to integrate self-learned reward information with social advice from a confederate partner. Separable learning rates were observed for reward and social information, correlating to activity in the ACC sulcus and ACC gyrus, respectively. Integration of reward-based and social information during the decision phase was correlated to activity in the vmPFC (Behrens et al., 2008). A role for the OFC in peer-related valuations has also been suggested based on a recent single-unit study in monkey by Watson and Platt (2012), in which subjects chose between receiving fluid rewards or viewing socially relevant images. Neurons recorded in the OFC consistently registered socially relevant information and signaled attentional duration toward social imagery (Watson and Platt, 2012), suggesting an important role for OFC in assessing conspecifics. While neither of the above tasks were competitive in nature, it is likely that the same prefrontal regions would be active in competitive tasks, when peer group characteristics (*g, y*) need to be assessed and integrated with resource information (*x*) to guide choice behavior. The higher the utility estimate that results from *U* = *x* + (*x* * (*a*_1_ * (*g* * *y*))), the higher the likelihood that a subject will choose to engage in competition.

Multi-factorial utility assessments continue in the outcome evaluation phase. Successful achievement of a goal following aggressive action should result in two further endowment effects on perceived utility: one stemming from effort expenditure (*e*), and one from continued peer interest (*a*_2_). Effort itself is generally aversive and can discount initial estimates of *x*, affecting choice behavior (Walton et al., 2007; Botvinick et al., 2009; Hillman and Bilkey, 2010, 2012). However, expended effort can also enhance perceived value of an outcome once achieved, in line with theories of cognitive dissonance and deservingness (Feather et al., 2011; Johnson and Gallagher, 2011). Rewards acquired after skill or effort assume higher subjective worth than if acquired via windfall or with little effort (Zink et al., 2004; Vostroknutov et al., 2012; Hernandez Lallement et al., 2013). For example, participants who exert high-effort to obtain monetary rewards are subsequently more averse to donating that money, vs. donating money gained by windfall (Hernandez Lallement et al., 2013).

The amount of tactical effort required (*e*) should therefore enhance perceived utility immediately after the contested-for outcome has been achieved: *U* = *x* + (*x* * (*a*_1_ * (*g* * *y*))) + *e*. When effort is required to obtain a reward, BOLD activity increases in the amygdala and striatum upon reward receipt, while OFC reward-related activity remains unchanged (Elliott et al., 2004; Zink et al., 2004; Katsyri et al., 2013). Importantly, as recently shown by Hernandez Lallement et al. (2013), the endowment effect of effort is dependent on the size of reward obtained. High-effort that results in high reward appears to have a positive endowment effect, and correlates to increased activity in the ventral striatum. However, high-effort that results in a low reward is a disagreeable situation, and correlates to increased activity in AI (Hernandez Lallement et al., 2013).

If a goal is successfully achieved following direct competitive aggression, perceived utility should also be enhanced by continued peer interest or desire for the contested-for outcome (*a*_2_, where 0 < *a*_2_ < 1), a subtle schadenfreude type endowment effect. Continued peer interest endowment (*a*_2_) may be modified by peer group composition (1 < *g* < 2), similar to what is proposed for *a*_1_, whereby *U* = *x* + (*x* * (*a*_1_ * (*g* * *y*))) + *e* + (*a*_2_ * g). Schadenfreude and its opposing partner envy are more likely to arise when fellow competitors (*g*) are self-relevant, salient conspecifics (Takahashi et al., 2009). BOLD activity in the ventral striatum and OFC correlate to self-reports of schadenfreude (McClure et al., 2004; Fehr and Camerer, 2007; Takahashi et al., 2009), and activity in the dorsal ACC to self-reports of envy (Takahashi et al., 2009). Ventral striatal activations are also noted in two-person tasks that are not explicitly competitive, but where monetary pay-out information is provided to both players at the end of each trial (Fliessbach et al., 2007). If person A's payout is higher than that of person B, striatal activity increases in person A and decreases in person B, independent of the actual financial amount being awarded (Fliessbach et al., 2007).

When resource valuation, pre- and post-competition, is viewed in this multi-factorial light, it helps to explain the “joy” of winning, or conversely the enhanced feelings of loss, unfairness or pain after losing. Whereas in non-competitive situations one might gain/lose a resource of *U* = *x*, in a competitive situation one gains/loses a resource of *U* = *x* + (*x* * (*a*_1_ * (*g* * *y*))) + *e* + (*a*_2_ * g). The joy of winning and pain of losing have recently been posited as single variables ρ_win_ and ρ_loss_ by van den Bos et al. (2013) and incorporated into a verifiable learning model. Herein a starting framework is proposed to account for the skewed utility estimates of contested-for resources, which would help to explain differences in motivated action based on the presence of a competitor and the animacy of that competitor. When humans or monkeys compete against conspecifics, compared to against a computer, they are more attentive and quicker to act (Washburn et al., 1990; Hosokawa and Watanabe, 2012; van den Bos et al., 2013). However, suboptimal action can often result; e.g., bidding approaches rational agent predictions when humans compete against computers, but characteristics of the Winner's Curse appear when humans play against other humans (van den Bos et al., 2008, 2013). Competing against a conspecific elicits greater neural activity in outcome valuation networks and the IAN as compared to competing against a computer (Zink et al., 2008), perhaps indicative of this multi-factorial utility assessment.

## Concluding remarks

In non-pathological conditions, competitive aggression is an instrumental behavior, used to achieve an outcome that aids in self-preservation. Inherently it is a selfish behavior, with a binary outcome for the individual: good or bad. Reduced in this way, it is intuitive that competitive aggression utilizes reward-based reinforcement learning systems in the brain. As competitive behavior neuroscience progresses, it will be important to test social and non-social choice tasks in the same participant, in the same session, to delineate any uniquely social computations. Moreover, highly salient, realistic resources should be included in the non-social choice tasks to ensure directional attention that is on par with the directional attention prompted by the prospect of winning. It will be important to parse temporal sequences of activation in terms of pre-choice, choice, and post-choice, and to examine functional coupling between reward networks and the IAN that may be predictive of competitive dispositions.

In part competitive aggression is a learned mode of behavior, repeated when it is reinforced. But it is also a behavior motivated by skewed utility estimates, which may account for some of the violations of associative learning that are commonly observed in competitive environments. A preliminary model has been posed herein to illustrate how, in competitive scenarios, peer group endowment effects act to artificially inflate perceived benefit of a resource in the decision phase [*U* = *x* + (*x* * (*a*_1_ * (*g* * *y*)))] and in the outcome evaluation phase [*U* = *x* + (*x* * (*a*_1_ * (*g* * *y*))) + *e* + (*a*_2_ * g)]. These skewed utility estimates can prompt competitive actions which are ultimately costly to the individual or group. Peer group endowment effects may be particularly strong in adolescence (Blakemore and Robbins, 2012), in corporate cultures (Malhotra et al., 2008), or more generally in contemporay society, where winning is often prioritized above all else. Thus, the first rule of aggressive competitive action should be cost-benefit analysis, but mindfully careful cost-benefit assessment at that.

### Conflict of interest statement

The authors declare that the research was conducted in the absence of any commercial or financial relationships that could be construed as a potential conflict of interest.
